# Real-world experience of full-thickness traumatic macular hole among young patients

**DOI:** 10.1186/s40942-024-00539-3

**Published:** 2024-02-21

**Authors:** Ragukumar Venugopal, Anthony Vipin Das, Brijesh Takkar, Michael W. Stewart, Raja Narayanan

**Affiliations:** 1 Department of eyeSmart EMR & AEye, L V Prasad Eye Institute, Hyderabad, Telangana India; 2 Indian Health Outcomes, Public Health, and Economics Research Center, L V Prasad Eye Institute, Hyderabad, Telangana India; 3 Anant Bajaj Retina Institute, L V Prasad Eye Institute, Hyderabad, Telangana India; 4 Department of Ophthalmology, Mayo Clinic Florida, Jacksonville, FL USA

**Keywords:** Traumatic Macular Hole, Electronic Medical records, Optical coherence tomography, Visual acuity, Vitrectomy

## Abstract

**Objective:**

To describe the demographics, clinical, and imaging characteristics, and visual outcomes in young patients with full-thickness traumatic macular hole (TMH).

**Methods:**

This retrospective hospital-based study included patients with full-thickness TMH who presented between August 2010 and June 2021. Demographic data, clinical findings, and imaging characteristics were extracted from an electronic medical record system. Regression analyses were performed to determine significant associations among variables and to identify predictors of visual outcomes.

**Results:**

144 (0.005%) patients among 2,834,616 were diagnosed with Full thickness TMH. The majority of them were male (89.58%; odds ratio [OR] = 6.71) and the holes were unilateral. The mean age at presentation was 23.37 ± 8.19 years. Ball were the most common cause of injuries (22.22%), followed by stick (14.58%) and firecracker (12.50%). The mean LogMAR visual acuity (VA) at presentation was 1.18 ± 0.72, with 25.69% of eyes having VA < 20/400. The mean minimum hole diameter was 619.34 ± 336.16 μm. Sub-retinal fluid was present in 44.44%, followed by intraretinal fluid in 34.03% of eyes. Macular holes closed after vitrectomy in 66.67% of eyes, with mean final VA of 1.07 ± 0.85. Baseline VA was a strong predictor of final VA (R^2^ = 0.677; *p* = 0.000168).

**Conclusion:**

Traumatic macular hole is a unilateral condition with significant visual impairment that is mainly seen in males during the third decade of life. Surgery is successful in most cases but improvements in VA are modest.

**Supplementary Information:**

The online version contains supplementary material available at 10.1186/s40942-024-00539-3.

## Introduction

Full-thickness traumatic macular hole (TMH) resulting from indirect trauma to the macula, can lead to severe visual impairment with associated limitations in function [1–3]. It is a distinct form of macular hole characterized by a full-thickness defect in the neurosensory retina involving the fovea typically due to blunt trauma to the eye [4–5]. Blunt trauma of various types, including sports-related injuries, motor vehicle accidents, or falls, are responsible for the majority of TMH [6]. Traumatic hole occur predominantly in young males during their second and third decades of life, and the incidence within this cohort ranges from 1% to 9% [7, 8]. Visual recovery from TMHs can be hindered by tissue damage from associated entities that include choroidal rupture, optic neuropathy, sub-retinal hemorrhage, retina tear, or retinal detachment [9, 10].

Tangential forces from a contrecoup injury, together with mechanical disruption of the thin foveal tissue, lead to the formation of a full-thickness hole [11]. Optical coherence tomography (OCT) precisely visualizes the macular hole, identifies vitreous traction, intraretinal cysts, foveal splitting, and other retinal features, and enables measurement of its dimensions [12, 13]. Surgery with pars plana vitrectomy (PPV), internal limiting membrane peeling, and gas tamponade can close the holes and improve visual acuity (VA) in most cases [9, 10].

There is limited data on the prevalence and demographic distribution of TMH in patients less than 40 years, so the purpose of this study is to define the real-world experience of TMH in young patients, to describe the typical findings on OCT, and to report the clinical course in affected patients presenting to a multi-tier ophthalmology hospital network.

## Materials and methods

### Study design, period, location, and approval

This retrospective hospital-based study included all patients presenting to L.V. Prasad Eye Institute located in India between August 2010 and June 2021 [14]. All patients gave a standard consent form for research and electronic data privacy at the time of registration in the clinic. The clinical data of each patient was entered into a browser-based electronic medical records system (eyeSmart EMR) by similarly trained professionals [15]. The study adhered to the Declaration of Helsinki and was approved by the Institutional Ethics Committee (Approval Number: LEC-BHR-R-10-23-1135).

### Inclusion criteria

**Fig. 1:**
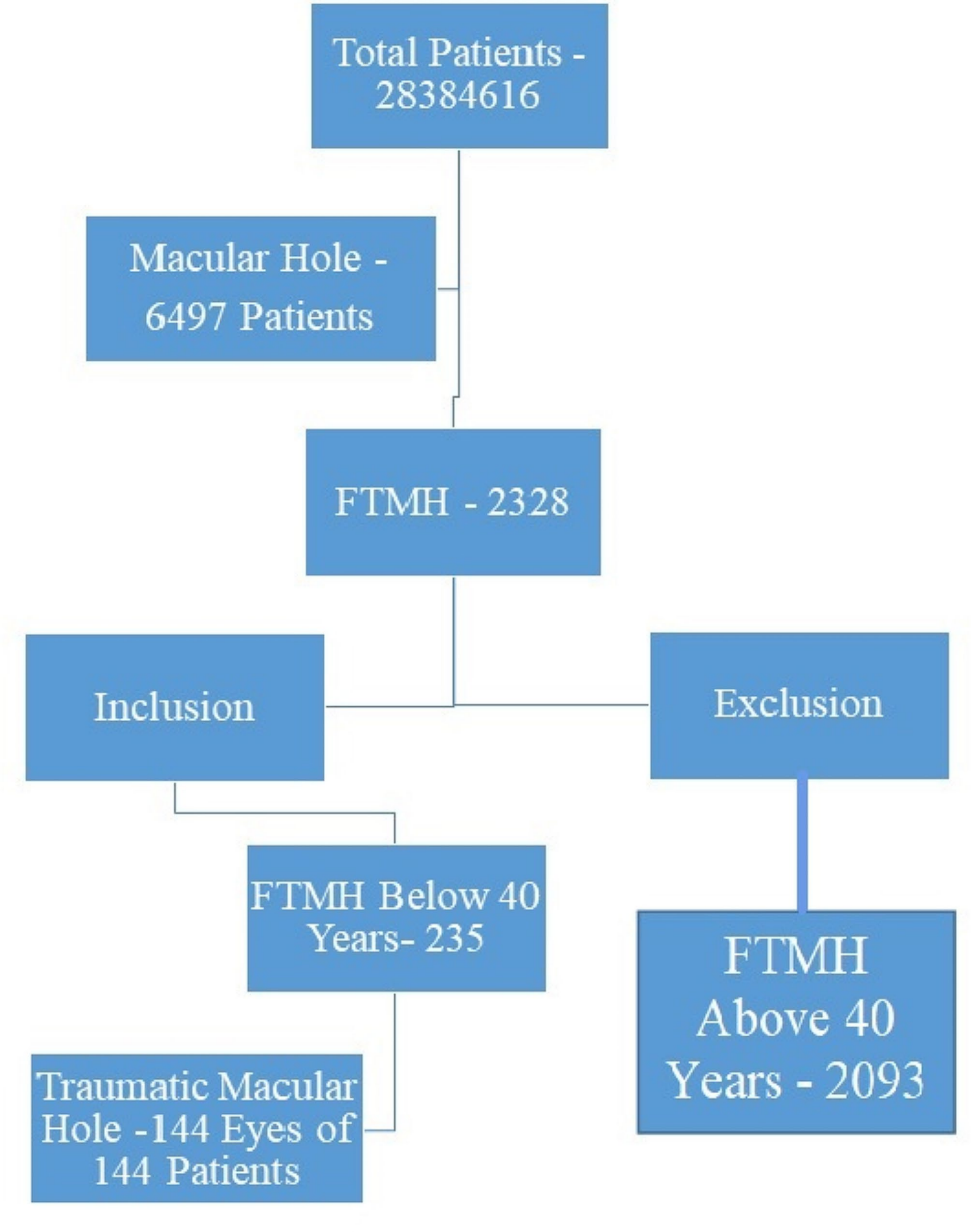
Cases included and excluded. FTMH - Full-thickness traumatic macular hole

Among a total of 2,834,616 new patients who presented to the tertiary and secondary centers during the study period, the EMR was queried for patients with an ocular diagnosis of macular hole in one or both eyes. A total of 6497 patients were identified using this search strategy. Among 6497 patients we found a total of 144 patients less than 40 years with a diagnosis of macular hole and a history of trauma to the eye were included in the study cohort (Fig. 1). We have included all type I closures as closed and type II and persistent non-closure of macular hole as not closed.

### Data retrieval and processing

The data of 144 patients and 1,250,115 non-macular hole patients included in this study were retrieved from the EMR database. The captured variables were patient demographics, clinical presentation, ocular diagnosis, OCT characteristics, and treatment information. Baseline OCT scans were analyzed to determine the minimum diameter and height of the macular hole. The excel sheet with the required data was then used for analysis using the appropriate statistical software. The VA was expressed according to the World Health Organization guidelines [16].

### Statistical analysis

Continuous variables were checked for normality using the Shapiro-Wilk test. Wilcoxon signed-rank tests were performed to assess the difference between the paired samples of initial VA and final VA, Chi-square was used to test for differences in demographic features between patients with TMH and the overall population. To test the relationships between the (final VA) and independent variables (gender, age, presenting VA, and associated ocular comorbidities), linear regression analyses were performed. Multiple logistic regression were performed for the closure of macular hole and its predictors. The odds ratio (OR) along with the 95% confidence interval of the predictor variables were calculated using R software (version 3.5.1).

## Results

### Prevalence

Among the 2,834,616 new patients who presented across the eye care network during the study period, 144 eyes of 144 patients were diagnosed with TMH, translating into a hospital prevalence rate of 0.005% or 5/million patients/ year. Our cohort had only one patient (< 1%) with high myopia.

### Age

The mean age of the patients was 23.37 ± 8.19 years and the median age was 23 (IQR: 18–30) years. The most common age group of the affected patients was 21 to 30 years [(*n* = 55; 38.19%); OR 3.71; 95%CI: 1.88–8.44] followed by 11 to 20 years [(*n* = 48; 33.33%); OR 4.57; 95%CI: 2.29–10.44]. The youngest patient being four years of age.

### Sex

Most patients were male 129 (89.58%) with sex being a significant risk factor for developing a traumatic FTMH (Male: 129/1,527,876; 0.0084%) vs female (15/1,306,740; 0.0011%) (*p = < 0.001*). The mean age among males 23.41 ± 7.97 (IQR: 18 to 30) was similar to that among females 23.06 ± 1.02 (IQR: 15 to 31). Odds of TMH were 6.71 times (95%CI: 4.07–11.95) higher in males as compared to females.

### Urban-rural distribution

Of the 144 patients with TMH, 68 (47.22%) were from an urban area, 59 (40.97%) were from a rural area and 17 (11.81%) patients presented from a metropolitan area. The overall prevalence of TMH disease in the urban community (0.0061%; 68/1,106,990) was higher than in the rural (0.0044%; 59/1,353,226) or metropolitan communities (0.0045%; 17/374,400) (*p* < *0.00001;* OR 1.28; 95%CI: 0.77–2.26; Table 1).

**Table 1:** Logistic regression analysis of factors associated with Traumatic macular hole (TMH)

Variables	Risk Factors	Co-Efficient	Odds Ratio	95% of Confidence Interval Lower Limit - Upper Limit	P value
Gender (Reference: Female)	Male	1.90	6.71	4.07–11.95	< 0.001
Age -Category (Reference: 0–10 Years)	11–20 Years	1.52	4.57	2.29–10.44	< 0.001
	21–30 Years	1.31	3.71	1.88–8.44	< 0.001
	31–40 Years	0.97	2.65	1.29–6.17	0.01
District Status (Reference: Metropolitan)	Urban	0.25	1.28	0.77–2.26	0.37
	Rural	0.13	1.14	0.67–2.03	0.65

### Laterality

Of the 144 patients with TMH disease, 82 (56.94%) of the macular hole were in the left eye and 62 (43.06%) were in the right eye.

### Nature of trauma

The following types of trauma were most frequently documented: ball (32; 22.22%), unspecified blunt trauma (38; 39%), stick (21; 14.58%), and firecracker (18; 12.50%) (Table 2). The mean time between injury and presentation was 22.54 ± 3.89 months, (IQR: 1–24) months and the average follow-up after diagnosis was 151.56 ± 310.79 (IQR: 0-135.75) days.

**Table 2:** Comparison of visual acuity between presentation and final visual acuity according to presenting history and complaints

Nature of Trauma	OD (62) n (%)	OS (82) n (%)	Visual Acuity at Presentation Mean ± SD	Visual Acuity at Final Visit Mean ± SD	P Value
**Ball**	15 (45.45)	18 (54.55)	0.92 ± 0.46	0.95 ± 0.58	0.67
**Unspecified Blunt Trauma**	18 (48.65)	19 (51.35)	1.19 ± 0.82	1.05 ± 0.82	0.09
**Stick**	11 (52.38)	10 (47.62)	1.43 ± 0.80	1.16 ± 0.67	0.02
**Fire-Cracker**	4 (22.22)	14 (77.78)	1.23 ± 0.67	1.15 ± 0.74	0.31
**Stone/Iron Projectile**	2 (20.00)	8 (80.00)	1.01 ± 0.48	0.84 ± 0.36	0.07
**Motor Vehicle Accident**	8 (66.67)	4 (33.33)	1.56 ± 1.04	1.24 ± 1.08	0.03
**Other injury (rope, rod, etc.)**	4 (30.77)	9 (69.23)	1.15 ± 0.48	1.11 ± 0.74	0.50

### Presenting visual acuity

The mean LogMAR baseline VA of affected eyes was 1.18 ± 0.72. Twenty (13.89%) eyes had mild visual impairment (> 20/70), 65 (45.14%) had moderate visual impairment (< 20/70 to 20/200), 22 (15.28%) had severe visual impairment (< 20/200 to 20/400), and 41 (25.69%) were worse than 20/400.

### Final visual acuity

**Fig. 2:**
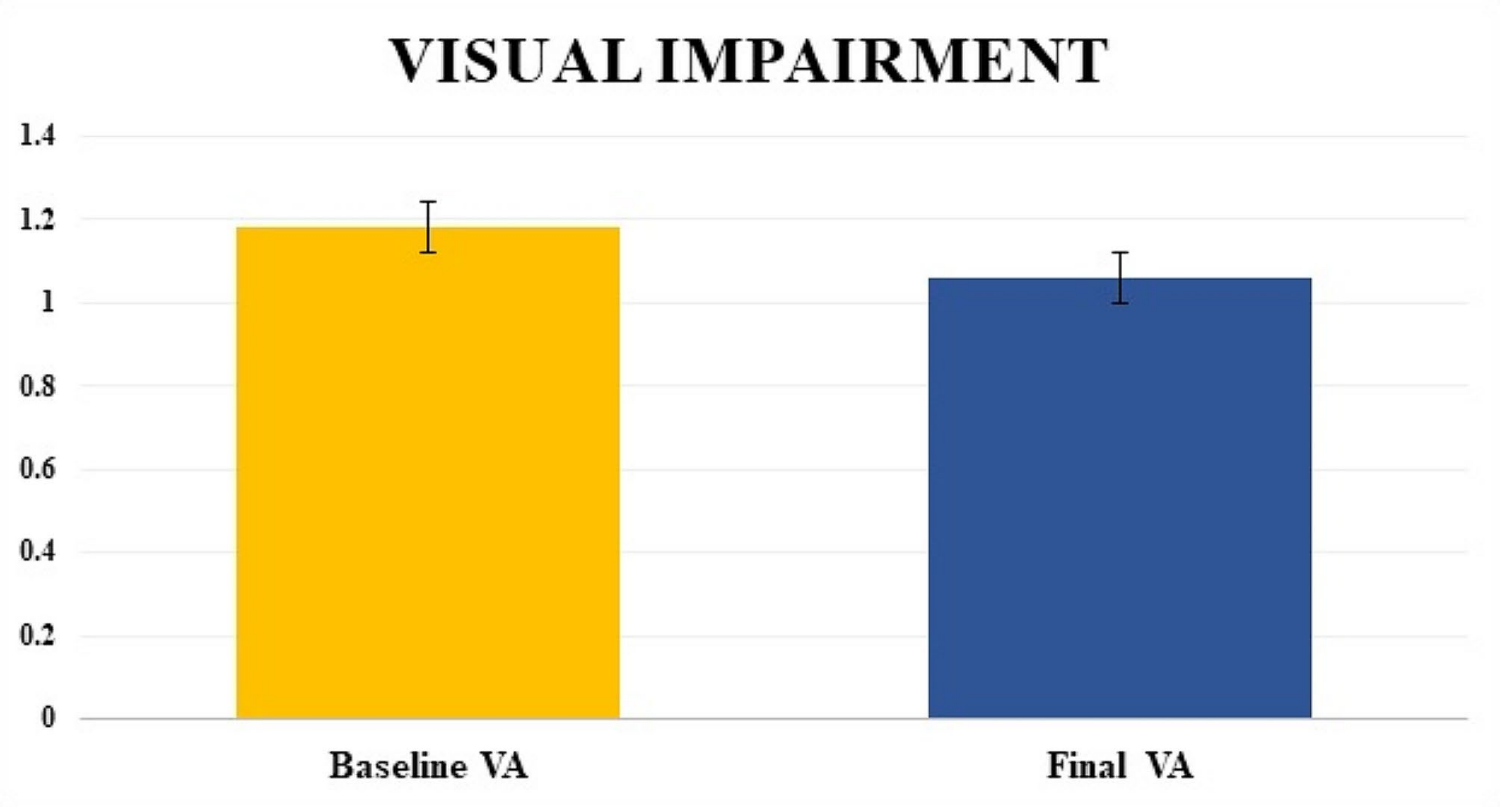
Comparison of LogMAR Mean and Standard error of visual acuity between diagnosis visit (Baseline Visual Acuity) and final visit (Final Visual Acuity) among overall eyes

The mean final VA of the affected eyes was improved to 1.06 ± 0.72 *(p = < 0.001)*. Initial VA is compared with final VA is shown in Fig. 2.

### OCT characteristics

**Fig. 3:**
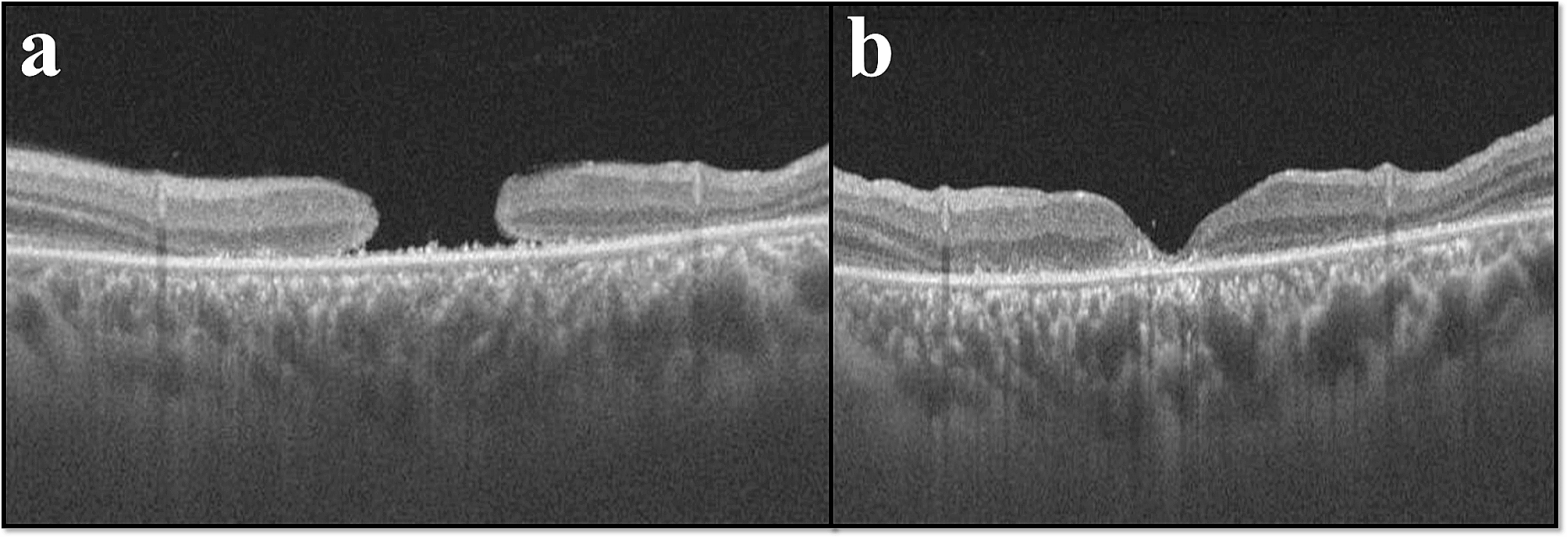
The right eye OCT of a 36-year-old male with a history of road traffic accident and CFCF vision with, a macular hole height of 257 µ, horizontal hole diameter of 1569µ, vertical diameter hole of 1444 µ, and minimum hole diameter of 810 µ. Minimal reflective spaces were present within the neurosensory retina in the fovea with loss of retinal tissue. The (b) frame shows successful closure following vitrectomy surgery with an interval of 40 days between surgery and the OCT imaging

The mean minimum hole diameter was 619.60 ± 336.16 μm (IQR: 389–817) and the mean height was 313.60 ± 178.48 μm (IQR: 215–389). Subretinal fluid was noted in 64 (44.44%) eyes, cystic edema in 53 (36.81%), detachment of margin in 28 (19.44%), and intraretinal fluid in 49 (34.03%) (Fig. 3). OCT characteristics of patients underwent PPV are shown in additional file 1.

### Retinal co-morbidities

Choroidal rupture was documented in 25 (17.36%) eyes, epi retinal membrane in 19 (13.19%) eyes, angle recession in 14 (9.72%) eyes, sub-retinal hemorrhage in 11 (7.64%) eyes, retinal detachment in 10 (6.94%) eyes, posterior vitreous detachment in nine (6.25%), retinal pigment epithelium rupture in seven (4.86%), bruch’s membrane rupture in six (4.17%) eyes, glaucoma in five (3.47%) eyes, and subluxation of the lens in two (1.39%) eyes.

### Factors influencing final visual outcome

Linear regression analysis showed that baseline VA was a predictor of the final VA (R^2^ = 0.67; *p* < 0.0001). (Table 3) and ball injuries were associated with poor final VAs.

**Table 3:** Factors influencing final visual outcome

Predictor	Co-Efficient	P Value
Gender	0.02	0.96
Diagnosis Age	0.00	0.99
Presenting Visual Acuity (LogMAR)	0.51	< 0.0001
Eyes Undergoing ILM Peeling Surgery	-0.12	0.38
Epiretinal Membrane	-0.11	0.61
Intraretinal Fluid	-0.02	0.90
Bruch’s Membrane Rupture	044	0.20
Posterior Vitreous Detachment	0.02	0.96
Sub Retinal Fluid	0.00	0.98
Sub Retinal Hemorrhage	0.00	0.33
Retinal pigment Epithelium Damage	-0.27	0.40
Glaucoma	-0.49	0.33
Retinal Detachment	0.08	0.68
Angle Recession	0.09	0.65
Choroidal Rupture	0.13	0.45
Subluxation of lens	1.01	0.07
Duration of Injury	0.00	0.39
Hole Height	0.00	0.70
Minimum Hole Diameter	0.00	0.05
Ball– Injury	0.13	0.45
Fire Crackers– Injury	0.29	0.24
Stick– Injury	0.05	0.82
Blunt Trauma– Injury	0.14	0.46

### Surgical treatment

**Fig. 4:**
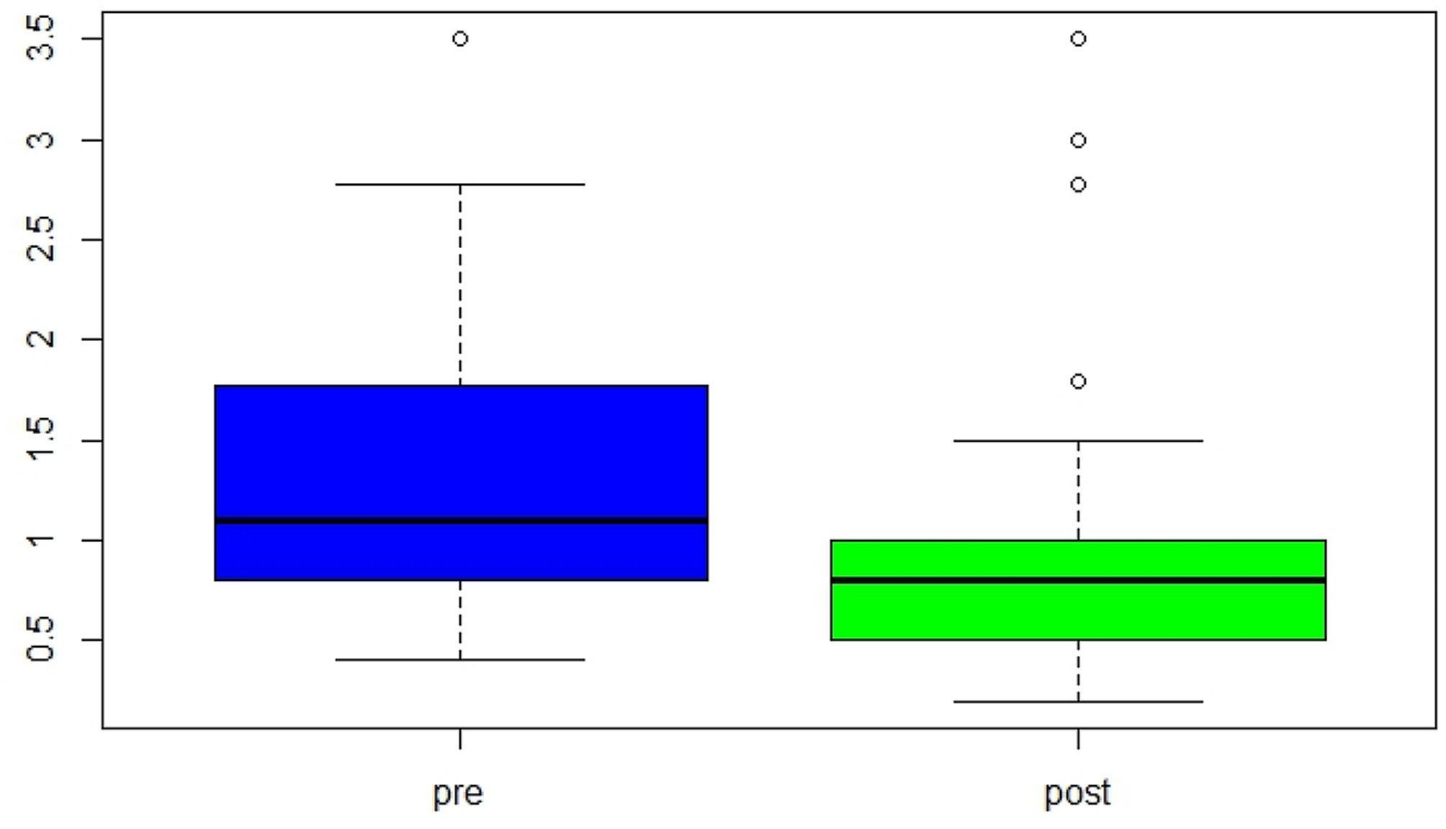
Comparison of the median, SD, IQR, and maximum value of visual acuity between preoperative (pre) and postoperative (post) among vitrectomy surgery eye

The pre-operative and post-operative characteristics of eyes that underwent surgery are shown in Fig. 4. Vitrectomy with internal limiting membrane (ILM) peeling was performed in 42 (29.17%) eyes, with closure in 28/42 (66.67%) eyes, failure to close in 7/35 (16.67%) eyes, and insufficient post-operative data from the remaining seven (16.67%) eyes. Spontaneous closure was noted in 17/91 (18.68%) eyes of patients with follow up. The mode of injury was not associated with closure of the macular hole (Table 4).

**Table 4:** Logistic regression analysis of factors associated with hole closure

Variables	Risk Factors	Co-Efficient	Crude Odds Ratio	95% of Confidence Interval	P value
				Lower Limit - Upper Limit	
**Gender**	**Male**	1.20	3.33	0.37–25.98	0.25
**Age**	**21–30 Yrs**	-0.81	0.44	0.08–2.71	0.36
	**31–40 Yrs**	1.20	3.33	0.47–67.68	0.30
**Mode of injury**	**Ball**	0.67	1.64	0.21–34.01	0.67
	**Fire Crackers**	-1.50	0.22	0.01–6.15	0.31
	**Stick**	-1.65	0.19	0.02–1.90	0.14
	**Blunt Trauma**	0.88	2.40	0.33–49.13	0.45
**Co-morbidities**	**ERM**	0.49	1.64	0.21–49.13	0.45
	**IRF**	1.50	4.50	0.81–35.68	0.10
	**SRF**	0.58	1.78	0.33–10.50	0.50
	**SRH**	-1.50	0.22	0.01–6.15	0.31

ERM– epiretinal membrane; IRF– intraretinal fluid; SRF– subretinal fluid; SRH– subretinal hemorrhage

Pre and post-operative comparison of VA to co-morbidities and complaints is shown in Table 5. In the 42 eyes that underwent vitrectomy, the mean baseline VA was 1.33 ± 0.80, and the final VA after vitrectomy was 1.07 ± 0.85 (*p = < 0.013).* 27 (64.29%) eyes had better vision, 5 (11.90%) eyes had stable vision, and 10(23.81%) eyes had worsening vision from the initial visit. Comparison of clinical parameters in patients with follow-up- PPV vs. Without PPV is summarized in Table 6.

**Table 5:** Pre and post-operative comparison of visual acuity with co-morbidities

Nature of Trauma	n (%) 42 Eyes	Comorbidities		Visual Acuity at Presentation Mean ± SD	Visual Acuity at Final Visit Mean ± SD
		SRH n (%)	Choroidal Rupture n (%)		
**Ball**	8 (19.50%)	1 (2.38%)	2 (4.76%)	0.75 ± 0.41	1.01 ± 0.88
**Unspecified Blunt Trauma**	9 (21.43%)	NA	NA	1.35 ± 0.77	0.99 ± 0.96
**Stick**	6 (14.29%)	NA	2 (4.76%)	1.81 ± 1.08	1.18 ± 0.79
**Fire-Cracker**	5 (11.90%)	NA	1 (2.38%)	1.56 ± 0.73	1.44 ± 0.98
**Stone/Iron Projectile**	3 (7.14%)	1 (2.38%)	NA	1.10 ± 0.70	0.63 ± 0.40
**Motor Vehicle Accident**	6 (14.29%)	NA	NA	1.54 ± 0.97	0.95 ± 0.94
**Other injury (rope, rod, chemical, etc.)**	5 (11.90%)	NA	NA	1.29 ± 0.65	1.20 ± 0.92

**Table 6:** Comparison of clinical parameters in patients with follow-up– PPV vs. Without PPV

Parameters	PPV– (*n* = 42)	Without PPV; With follow up - (*n* = 49)	p - Value
	Mean ± SD	Mean ± SD	
**Age**	24.48 ± 7.93	23.61 ± 8.07	0.609
**Initial Vision**	1.33 ± 0.80	1.10 ± 0.70	0.130
**Final Vision**	1.07 ± 0.85	0.98 ± 0.64	0.469
**Hole Size**	637.46 ± 317.02	572.93 ± 404.56	0.196

**PPV-** Pars Plana Vitrectomy; *n* = 53 were lost to follow-up and not included in the table

## Discussion

The purpose of this study was to determine the prevalence, demographic profile, OCT characteristics, and visual outcomes of patients under the age of 40 years with TMH who presented to a multi-tier hospital network. The overall prevalence of TMH disease was 0.005% among all newly evaluated patients between 2010 and 2021 (an 11-year period).

Among patients with full-thickness macular holes, between 1 and 9% may be due to trauma [7]. Johnson et al. [10] observed that 80% of patients with TMH were males with a mean age of 23 years (range: 8 to 36 years). Similarly, we found that most patients were male with a mean of 23.38 years, and TMH was more prevalent in urban patients. García-Arumí J et al. [17] found that the common causes of TMH were ball injuries or physical trauma with jabs or kicks. We also found that most patients had a history of injuries due to balls, unspecified blunt trauma, firecrackers, and sticks.

OCT is indispensable for making the diagnosis of TMH [18]. In our study OCT was performed in (55.56%) of eyes, with the most common associated findings being sub-retinal fluid, intra-retinal fluid, choroidal rupture, and epiretinal membrane. Depending on the macular anatomy, patients usually present with VAs between 20/30 and 20/400 [10, 18]. The mean LogMAR VA in our patient was 1.18 (20/320) at baseline, which improved by one line to 1.06 (20/250; *p* < *0.001*) at the final follow-up, suggesting that surgical interventions produced statistically significant improvements. Furthermore, the regression analysis showed that baseline VA was significant predictor of the final visual outcome, and it also indicates that approximately 67.7% of the variability in the last VA can be explained by the independent variables included in the model. This suggests that the model has a moderately good overall fit or moderate level of explanatory power. Other independent variables, including gender, diagnosis age, and associated retinal co-morbidities, did not show a significant association with the final VA. These findings suggest that baseline VA is a crucial factor in determining the overall visual outcome in TMH patients. However, as the presentation was late in our follow-up in most patients, this may not hold true for early or new-onset TMH.

Vitrectomy with ILM, air-fluid exchange, and face-down positioning is the most important treatment option for eyes TMH [13]. Hou J et al. [19] found the mean LogMAR VA improved from 1.06 to 0.84 after vitrectomy. In our study, VA improved by an average of two lines after surgery and 67% of treated holes closed successfully. Ball injuries had a poorer prognosis compared to other etiologies. Importantly, only 29% of our patients underwent vitrectomy surgery. Surgery was performed at the discretion of the examining physician and was based on an assessment of the likelihood of visual benefit. Many of the eyes had co-existent chorioretinal damage that was believed would prevent meaningful improvement, so surgery was not offered. Additionally, many patients elected to not undergo surgery for personal reasons. Improvement in VA after macular hole surgery is usually best when surgery is performed within the first six months, and the long mean time to diagnosis in this cohort was an important factor that limited the number of patients undergoing surgeries. This underscores the importance of early diagnosis for the optimal management of these patients.

The strength of the study includes a comprehensive analysis of a large cohort of patients under 40 years of age. Our study also provides real-world evidence of surgical interventions in TMH.

The limitation of our study includes the drawback of a retrospective study which includes incomplete or missing data, potential selection bias, and consistency of the recorded information. In this study we provided a comprehensive demographic and clinical representation. Therefore, we included all patients in the initial analysis.

In conclusion, this study provides important insights into the clinical presentation and visual outcomes of patients with TMH. The findings highlight the association of TMH with certain demographic factors, modes of injury, and the effectiveness of surgical intervention in improving VA. These contribute to a better understanding of TMH and may guide clinical management of such cases.

### Electronic supplementary material

Below is the link to the electronic supplementary material.

Supplementary Material 1

## Data Availability

Data cannot be shared publicly because of confidentiality agreements. Data are available from the LVPEI Institutional Data Access/ Corresponding author for researchers who meet the criteria for access to confidential data.

## Abbreviations

TMH: Traumatic Macular Hole
VA: Visual Acuity
OCT: Optical Coherence Tomography
OR: Odds Ratio
ILM: Internal Limiting Membrane
