# Supplementary material for: Real-world experience of full-thickness traumatic macular hole among young patients
Source: Int J Retina Vitreous. 2024 Feb 21;10:20. doi: 10.1186/s40942-024-00539-3 (PMC10882818; doi:10.1186/s40942-024-00539-3)
Supplement: Supplementary file 1 — Supplementary Material 1 [file 40942_2024_539_MOESM1_ESM.docx]

**Additional file 1: OCT Characteristics of Patient Underwent PPV**

| **PPV – OCT Characteristics(n=42)** | **n** | **%** |
| --- | --- | --- |
| **SRF** | 21 | 50.00% |
| **IRF** | 23 | 54.76% |
| **ERM** | 8 | 19.05% |
| **Cystic edema** | 22 | 52.38% |
| **Marginal Detachment** | 10 | 23.81% |
| **PVD** | 3 | 7.14% |

PPV – Pars Plana Vitrectomy, SRF- Subretinal fluid, IRF – intraretinal fluid, ERM – epiretinal membrane, PVD – posterior vitreous detachment
